# From catwalk to kitchen: A qualitative study of luxury fashion companies’ diversification into hospitality and restaurant sector

**DOI:** 10.1371/journal.pone.0350846

**Published:** 2026-07-01

**Authors:** Simone Luongo, Fabiana Sepe, Elena Lupolo, Giovanna Del Gaudio

**Affiliations:** 1 Faculty of Economics and Law, Department of Management and Economics, Università Telematica Pegaso, Centro Direzionale, Isola F2, Napoli, Italy; 2 Department of Economics, Management, Institutions, University of Naples Federico II, Naples, Italy; Sapienza University of Rome: Universita degli Studi di Roma La Sapienza, ITALY

## Abstract

This study examines the strategic diversification of luxury fashion brands into the hospitality and restaurant industry, with a particular focus on sustainability. The objective is to explore the motivations behind this cross-industry expansion, the strategies employed and the sustainable practices integrated into such ventures. A qualitative research design is applied, drawing on semi-structured interviews with stakeholders from leading luxury fashion companies. This approach allows for an in-depth understanding of how experiential branding, strategic decision-making and sustainability are embedded within hospitality initiatives. The findings reveal that luxury brands enter the hospitality sector to enrich the brand experience, strengthen customer engagement and foster innovation in sustainability. Sustainable practices are identified both in operational management and in customer-facing activities, though the extent of integration varied across cases. The results underscore the dynamic relationship between experiential branding and sustainability, highlighting opportunities for competitive differentiation as well as challenges in aligning brand identity with new industry practices. This research contributes to the growing literature on luxury brand management and diversification by demonstrating how sustainability influences experiential strategies in cross-industry contexts. The originality of this paper lies in its integrated view of luxury branding, diversification and sustainability, showing how fashion companies leverage hospitality ventures to reinforce brand value and connect with socially conscious consumers.

## Introduction

Corporate diversification is a crucial strategy for luxury brands, ensuring growth, resilience and long-term sustainability in an increasingly competitive and dynamic global environment. While diversification is used to be mostly limited to product line extensions, it currently includes a wide range of strategic choices, including geographic expansion, business model innovation and customer segmentation [1,2].

Many luxury fashion brands have expanded their offerings to include‌‌ lifestyle services such as hospitality and interior design to strengthen their brand image and foster deeper emotional connections with consumers [3]. At the same time, brands have adapted to market changes by introducing more affordable product lines, entering emerging markets (e.g., China) and adopting corporate philanthropy initiatives to build social legitimacy and access new market segments [4].

Alternative business models, such as the luxury second-hand market, reflect a growing focus on sustainability and the preferences of younger, environmentally conscious consumers [5].

Companies tend to diversify into sectors with similar operational logics; transitions into entirely new sectors are riskier [6]. In this context, the key to success lies in adapting internal competencies without compromising brand authenticity [7,8].

This is particularly relevant when analysing the expansion of luxury brands into the restaurant and hospitality sectors. Iconic brands such as Gucci, Dior, Chanel, Versace and Armani have opened cafés and restaurants that combine culinary experiences with the visual and stylistic language of the brand, as well as hotels that offer a similar experience. Representative examples include Dior Café, Gucci Osteria in collaboration with Michelin‑star chef Massimo Bottura, the Beige Alain Ducasse by Chanel in Tokyo, Palazzo Versace in Dubai and the Armani Hotel in Milan and Dubai [9]. Therefore, these experiences function as true emotional shop-in-shops, where consumption becomes a form of storytelling that fosters a sense of belonging.

The extensions represent an evolution of experiential branding. They enable consumers to live the brand directly and multisensory, thereby strengthening loyalty, emotional attachment and perceived quality [10]. Furthermore, they increase dwell time, the amount of time spent engaging with the brand, acting as catalysts for engagement and narrative coherence between products and services [11].

We adopt fine dining as a privileged analytical lens because it constitutes a highly orchestrated, multisensory service system in which brands can enact symbolic meanings as embodied experiential narratives and test experiential coherence [12]. Moreover, haute cuisine represents a high-fit, high-signal arena for brand extension: meta-analytic evidence shows that extension success depends jointly on perceived fit and parent-brand equity, while studies of luxury fashion-brand restaurants and cafés link perceived quality and luxury value to loyalty toward both the extension and the parent brand [13,14]. Taken together, fine dining offers a stringent empirical context for examining how brands sustain identity across service touchpoints while generating measurable effects on consumer response and brand equity [15,16].

In addition, while luxury has traditionally been associated with excess, indulgence and waste [17,18], a new paradigm is emerging in which sustainability is becoming a strategic priority. This is being driven by growing consumer awareness of environmental and social issues, as well as the increased global exposure of the luxury sector [19,20].

In response to these dynamics, luxury firms are increasingly embedding Corporate Social Responsibility (CSR) into their business strategies as a structured approach to address social and environmental challenges while pursuing economic performance [21,22]. CSR is progressively adopted as an integrative framework that guides corporate behaviour, enabling companies to balance profitability with responsible practices and to generate value for a broad range of stakeholders [23].

This value creation extends beyond shareholders to include employees, suppliers, local communities, institutions and consumers, fostering long-term relationships based on trust, fairness and transparency. [24]. From this perspective, the analysis of social sustainability becomes central, encompassing dimensions such as employment generation, quality of working conditions, skills development and the socio-economic impact on local communities [25].

In the luxury sector, particularly within hospitality and fine dining activities, these dimensions translate into the valorisation of human capital, support for local supply chains and the promotion of inclusive practices that contribute to territorial development.

Flagship stores and multisensory spaces thus become critical platforms for demonstrating environmental commitment, while digital content, such as artisanal videos shared on social media, reinforces experiential engagement and ethical identification [26]. Emblematic examples include sustainable luxury resorts involved in conservation projects, as well as the growing demand for authentic, locally sourced, sustainable food products [27]. Despite these opportunities, achieving sustainability in luxury tourism and gastronomy is challenging. Tourism activities can be particularly energy and water intensive and offering experiences in fragile ecosystems raises ethical and environmental concerns [28,29]. Accordingly, although the concept of sustainable luxury is gaining attention, there is still a lack of systematic, in-depth studies investigating the relationship between luxury experiences, particularly in hospitality, fine dining and multidimensional sustainability [12,30]. This gap is particularly evident given the growing demand from consumers for responsible practices and the need for luxury brands to balance their signature exclusivity and experiential offerings with emerging ethical and environmental concerns.

To date, there is a lack of studies that have thoroughly examined how such experiences can be designed in a sustainable manner without compromising the exclusivity and quality that define the essence of luxury.

Considering this scenario, the present study aims to address this gap by examining the strategic rationales, operational choices and experiential branding practices adopted by luxury fashion brands when they diversify into the hospitality and fine dining.

Attention will be paid to coherence between core brand identity and new experiential offerings and to mechanisms for integrating sustainability principles while maintaining a perception of luxury.

In line with the research objective, the study aims to address the following research question:

*RQ: How do luxury fashion brands strategically integrate sustainability principles into their diversification into the fine dining, while ensuring experiential coherence with their core brand identity?*

To reach the research objective, this paper adopts a qualitative methodological approach based on semi-structured interviews. The sample consists of eight key informants selected for their direct involvement and expertise in the field. Participants included professionals from top management, F&B, R&D, sustainability and marketing office of fashion companies involved in hospitality ventures.

The manuscript is structured as follows: first, the theoretical background is introduced, followed by an explanation of the methodology. The results are then presented and discussed. Finally, conclusions, implications, limitations and future research directions are outlined.

## Theoretical background

### Corporate diversification in luxury sectors‌‌

For luxury firms, diversification has evolved beyond merely expanding product lines; it encompasses various strategic dimensions to adapt to a dynamic global market and changing consumer preferences [31]. Historically, luxury fashion retailers have broadened their product portfolios to include a wide array of lifestyle products, such as hospitality, driven by the dual goals of increasing profit and strengthening their brand image and awareness [32,33]. This type of diversification involves strategic choices in product design, sourcing and overall portfolio composition.

Some brands have entered lower-priced categories, targeting middle-class consumers as a response to market shifts [31]. Geographic or market diversification is also crucial. Luxury retailers have significantly expanded their physical presence in China, including national and provincial cities, necessitating localized marketing strategies to accommodate the country’s cultural and economic diversity [3]. Corporate philanthropy serves as a strategic tool, enabling market entry by fostering stakeholder goodwill and alleviating external pressures [4]. The sector has also seen significant diversification in business models and distribution channels. The rise of the second-hand luxury market is a clear example, allowing brands to attract new customer segments and maintain relevance [5]. This shift underscores the importance of marketing and supply chain diversification, particularly toward environmentally and ethically conscious younger generations such as Gen Z and Millennials [31]. Operational diversification, especially in supply chains, has become increasingly vital for wholesale apparel firms. To ensure resilience amid global disruptions, firms have implemented supplier diversification strategies involving geographic, structural and logistical adaptations to reduce safety stock, stabilize demand and improve recovery times [34].

Despite its advantages, diversification also presents notable challenges. Firms often face shortages of human resources, managerial skills and effective marketing and operational strategies [2]. Unrelated diversification offers a buffer against sector-specific downturns but carries increased risk. A critical consideration for luxury firms is the potential conflict between short-term financial gains and long-term sustainability. Firms pursuing diversification might prioritize immediate profitability, which could sometimes clash with the long-term benefits of initiatives like corporate philanthropy [4]. Product diversification, particularly for family firms, can pose a threat to socioemotional wealth, as it may be seen as a departure from the brand’s heritage [1].

A significant challenge is knowledge transferability. Past research indicates that first-order knowledge, gained through learning-by-doing in manufacturing, has low transferability across industries, reducing the likelihood of diversification into sectors with different learning dynamics [35,36]. Conversely, firms are more prone to diversify into sectors where their second-order knowledge (knowing how to learn from learning by doing) is applicable, typically in industries with similar learning by doing rates [6].

In the contemporary luxury landscape, sustainability and innovation are no longer optional but strategic imperatives. Diversification strategies are increasingly intertwined with ethical conduct, technological advancement and local sourcing [31]. The growing demand for ethical practices has fueled sustainable models, such as second-hand luxury, highlighting their role in maintaining brand relevance [5]. Therefore, diversification in the luxury sector is a multifaceted strategic approach driven by evolving markets and consumer expectations. It requires careful management of market entry, product portfolio extension, business model innovation, resource allocation and knowledge transfer [1,6].

### Sustainable luxury and experiential branding

For luxury fashion brands, extending into the hospitality and restaurants sectors is an experiential marketing strategy that fosters emotional connections with consumers, reinforces brand identity and enhances brand equity [37]. Beyond financial gains, such diversification fosters consumer-brand relationships and generates brand admiration, especially when associated with stakeholder well-being and sustainable initiatives [38].

Nowadays, the global luxury industry includes a wide range of goods, services and experiences, such as fashion, automobiles, travel and gastronomy, with a significant global impact [15]. Luxury is traditionally associated with superior quality, uniqueness, craftsmanship and enduring value [37]. Experiential branding reinforces this by creating immersive experiences that build brand mystique and social aura [10,39,40]. Curated events and refined retail environments aim to educate consumers on the social value of goods, fostering appreciation and intimacy with the brand [41]. Sensory marketing plays a pivotal role, engaging consumers on a multi-sensory level and promoting emotional attachment and loyalty [36].

The inherent qualities of luxury, such as durability, timelessness and superior quality, align well with sustainability, promoting longer-lasting products and prudent resource use [13,42]. The scarcity often associated with luxury products may also indirectly contribute to more responsible consumption [17]. Furthermore, high prices in the luxury sector, by limiting demand, could contribute to conserving natural resources [15].

However, significant tension persists. Consumers often perceive sustainable luxury as less aesthetically appealing or desirable, especially when visual elements are prioritized [11,43]. This is termed the “fallacy of clean luxuries”, where consumers incorrectly assume luxury goods have minimal negative environmental or social impacts [44].

In addition, empirical studies suggest that brand prestige and luxury experiences can diminish the positive impact of sustainable product launches on consumer attitudes [45,46]. Products such as clothing made from recycled plastic may be perceived as inconsistent with luxury values [47].

The mass-market expansion of luxury brands through low-cost production and high-volume strategies [3] also undermines the sector’s compatibility with sustainability, raising ethical concerns about overconsumption and social inequities [14,42]. To address these challenges, brands must prioritize personalized experiences and transparent communication [13,48].

In this context, the emerging concept of quiet luxury offers a useful perspective through which to reconcile the tension between sustainability, aesthetic desirability and luxury value. Rooted in the broader literature on inconspicuous luxury consumption, quiet luxury refers to a form of luxury that prioritizes superior quality, craftsmanship, durability and aesthetic restraint [49,50].

This approach aligns luxury value creation with subtle forms of distinction, privileging cultural and experiential capital over conspicuous display.

From a sustainability perspective, quiet luxury provides a partial solution to the perceived conflict between luxury and ethical responsibility [51].

By prioritizing timeless design, long product lifecycles and refined materiality, quiet luxury aligns with principles of responsible consumption while preserving symbolic value and exclusivity [52]. Crucially, it also redefines luxury communication strategies, prompting brands to adopt more subtle narratives and experiential cues.

While luxury brands have historically been opaque regarding their practices [15,34], increasing demand for transparency requires them to disclose supply chain and sustainability efforts to avoid greenwashing [52,53].

Considering this scenario, especially in the hospitality and restaurant sector, Environmental, Social and Governance (ESG) attributes are gaining traction as key elements in brand management and consumer choice [9]. Effective communication of environmental initiatives is crucial to mitigate skepticism and enhance brand credibility [53]. For example, in the restaurant industry, the adoption of intelligent automation is transforming service roles. While mechanical tasks like order-taking are easily automated, more intuitive and empathetic tasks remain challenging, necessitating a redesign of service roles [54]. Some scholars argue that luxury brands should “whisper” rather than “shout” about their sustainability credentials to maintain exclusivity [15,55]. Moreover, several scholars support this by suggesting that staging experiences can bridge the attitude-behavior gap more effectively than traditional advertising [56,57]. Luxury includes tourism and gastronomy, offering fertile ground for sustainable experiential branding. Examples include eco-resorts supporting conservation and growing interest in sustainable, locally sourced luxury food products [58,59]. Therefore, it is vital to explore how to deliver sustainable luxury experiences without compromising perceived exclusivity, through resource efficiency, architectural design and effective communication. Luxury’s leadership in sustainability and innovation can trigger a trickle-down effect across mainstream markets, legitimizing sustainable luxury as a distinct research field [13,60].

### Brand extension into hospitality and restaurant sectors

The evolving landscape of luxury consumption has led luxury fashion brands to increasingly diversify beyond their traditional product lines and venture into the hospitality and restaurant sectors [9]. These strategic moves aim to offer authentic, immersive luxury experiences beyond physical products [15]. By captivating consumers with experiential elements, these extensions seek to enhance brand strength, build long-term customer relationships and diversify risks and revenue streams, contributing to business stability [61,62]. Luxury firms frequently collaborate with renowned chefs to maintain high-quality associations consistent with their core brand image [63]. Geographically, these initiatives are predominantly found in Asia and Europe, with Asian consumers, particularly drawn to their exotic value [64]. Consumer perceptions and motivations are critical to the success of these ventures. Existing literature highlights that perceived quality significantly influences perceived luxury value, which in turn drives customer loyalty, revisit intentions and recommendation behaviors for both the fashion brand and its hospitality and restaurant extension [65,66].

Key drivers of brand equity, including brand image, perceived quality and brand loyalty, are crucial in predicting customers’ post-consumption behavioral intentions. Specifically, materialism, brand image and perceived quality positively influence the intention to revisit the same luxury fashion-brand restaurant or café [15,67]. Specifically, uniqueness, brand loyalty, brand image and perceived quality predict the intention to visit other luxury fashion brand ventures. Furthermore, brand loyalty, brand image and perceived quality are key factors influencing the intention to purchase products of the same luxury brand after dining at their associated restaurant or café [14].

However, loyalty can yield complex outcomes. Strong brand loyalty may paradoxically reduce willingness to try other luxury F&B experiences [68]. Similar patterns emerge in fast fashion, where loyal consumers may react negatively to brand extensions into unrelated categories [69]. The perceived fit between the parent brand and the extension plays a crucial role. A negative fit can create undesirable associations, potentially harming the parent brand’s image [13,70].

This double-edged sword effect implies that while diversification can attract attention, clear and consistent communication is vital to prevent distrust and ensure effective consumer decision-making.

### Methodology

This study employs a qualitative methodological approach to investigate the complex phenomenon of luxury fashion brands diversifying into the hospitality and restaurant sector, with a particular focus on the fine dining, also considering the strategic integration of sustainability principles. Given the exploratory nature of this research and the nuanced dynamics involved in cross-industry diversification, a qualitative design was deemed most suitable for providing robust and in-depth understanding of the strategic rationales, operational choices and experiential branding practices adopted by luxury fashion firms. Indeed, this approach allows for a rich exploration of an emerging topic where existing theoretical frameworks may not fully explain the intricate interdependencies and evolving practices [71,72].

The research relies on semi-structured interviews with key informants. This method is suitable for its ability to provide rich, retrospective accounts and detailed insights into the perceptions, experiences and strategic decisions of individuals directly involved in this cross-industry expansion. Semi-structured interviews offer a flexible yet focused research method, allowing interviewees to elaborate on their perspectives while ensuring coverage of key research areas [73,74].

### Research design and sampling strategy

The study focuses on luxury fashion brands that have strategically diversified into the hospitality and restaurant sector. The selection of informants was critical to gaining a comprehensive understanding of this multifaceted phenomenon. Consequently, we employed a purposive sampling strategy, selecting participants based on their direct involvement and expertise in the field, which was crucial for an in-depth exploration of this niche area.

This targeted approach ensured that the sample consisted of individuals with specific and relevant expertise regarding luxury brand diversification into hospitality and restaurant industry. To gather a comprehensive and nuanced range of insights, snowball sampling was employed: initial participants, recognized experts in either the fashion or hospitality sectors, were asked to recommend other professionals with direct experience in luxury brand extensions.

This method was particularly effective for reaching high-profile informants who may not be easily accessible through conventional recruitment strategies. The starting point was the Brand Finance Apparel 2024 ranking, which lists the top 50 fashion brands globally in terms of brand value. From this list, we identified the fashion brands that have implemented diversification strategies into the hospitality and restaurant sector, resulting in a sample of 10 companies. Out of these 10, 3 did not respond and 2 declined to participate in the study. The final sample included 5 companies and a total of 8 key informants from the following business areas: top management, F&B, R&D, sustainability and marketing office. All interviewees are directly involved in hospitality ventures, particularly in fine dining, launched by fashion companies.

Prior methodological literature suggests that in homogeneous and elite samples, thematic saturation can be achieved with a relatively limited number of interviews [75]. In line with this, after eight interviews across five companies, recurring patterns and themes consistently emerged, indicating that further data collection was unlikely to yield substantially new information.

Importantly, interviewees were selected both from the fashion industry and the hospitality and restaurant sector, ensuring a well-rounded perspective on the phenomenon of cross-sector diversification. This diverse range of roles ensured that we captured perspectives from various functional areas, providing a holistic view of the motivations, implementation strategies and challenges raised in the diversification process. Moreover, having varying rankings ensured sample heterogeneity.

The selection process aimed for a balance of perspectives across different brands and roles to ensure a rich and varied dataset. Initial contact was made via professional channels (e.g., LinkedIn, company’s official website), outlining the study’s focus and inviting them to participate.

The willingness of these high-level professionals to participate underscored the relevance and emerging importance of this research area.

### Data collection

Data collection was conducted through semi-structured interviews, allowing for an adaptive exploration of the research question, in the period from February to April 2025.

Each interview followed a guide of open-ended questions designed to explore key areas such as motivations for diversification, strategies for brand integration, approaches to sustainability, operational challenges and the perceived impacts of these ventures. This open-ended design ensured that the scope of analysis was not constrained by pre-conceived categories.

Interviews were conducted in English, in a manner that maximized convenience and participation for the key informants. This included conducting interviews via online video conferencing platforms or by phone, depending on the interviewee’s preference and schedule.

This flexibility proved essential in securing participation from high-level professionals whose demanding schedules often preclude extensive in-person meetings. Despite the inherent lack of visual cues in some phone interviews, the semi-structured and open-ended nature of the discussions proved a natural and complementary fit, ensuring the consistency and richness of the collected data.

The lenght of each interview varied, allowing for in-depth discussions that typically lasted between 30–60 minutes. Before each interview, the interviewer provided a clear explanation of the study objectives, the voluntary nature of participation and the anonymization procedures. Verbal informal consent was obtained prior to starting each interview. Because interviews were only conducted when the participant explicitly agreed to proceed, no written or recorded documentation of the consent process was collected. All interviews were anonymized at the transcription stage and no identifying personal information was collected at any point. Considering the nature of the present study, that is not related to non-clinical or minimal-risk research in the social sciences, it does not require approval from an IRB or Ethics Committee. For this reason, IRB approval was not sought. The study adhered to standard ethical principles for research involving human subjects.

The key details about the interviewees are presented in Table 1.

**Table 1:** Interviews summary.

Label	Business area	Company headquarter location	Diversification in the same country of headquarter	Length of interview
R1	Top management	Italy	No	30 min.
R2	F&B	Italy	No	60 min.
R3	Sustainability office	USA	Yes; abroad	60 min.
R4	R&D	France	Yes; abroad	50 min.
R5	F&B	Italy	Yes; abroad	45 min.
R6	Marketing office	Italy	Yes; abroad	45 min.
R7	F&B	Italy	Yes; abroad	30 min.
R8	Marketing office	USA	Yes; abroad	30 min.

Source: Authors’ own elaboration.

To enhance the reliability and validity of our findings, the primary data from the interviews were complemented by secondary data sources. These supplementary materials included public statements from the luxury brands, press releases, company websites and articles from specialized gastronomic and fashion media. This integration of secondary data served multiple purposes: it helped to contextualize the interview insights, provided background information on the brands and their initiatives and allowed for cross-verification of information, thus mitigating potential biases and strengthening the credibility of our qualitative research findings.

To strenghten the interpretive accuracy, after each interview we prepared a brief participant summary that was later used for member checking. At the same time, we collected secondary sources (press releases, corporate websites, trade media) to contextualize and crossverify interview accounts, laying the ground for methodological triangulation reported in the next section.

### Data analysis

The data analysis process was systematic and iterative, designed to uncover emergent themes and patterns from the rich qualitative data. It involved two sequential phases, drawing inspiration from established qualitative research methodologies.

The process followed the Grounded Theory approach, that involves an inductive method in which general concepts and interpretative categories are constructed from individual events, situations and participants’ points of view [76]. This process allowed themes and patterns to emerge directly from the data, without the constraints of pre-existing frameworks or predefined categories [77,78]. Nonetheless, we also used literature-derived statements to construct the interview protocol (S1 Appendix).

The first phase focused on familiarization and initial coding. Following transcription of all interviews, the research team engaged in multiple discussions and readings of the transcripts to gain a deep familiarity with the research content [79]. During this phase, recurring expressions and significant statements from the interviews were identified and coded into “first-order concepts”. These initial codes represented the direct expressions and empirical observations derived from the informants’ narratives. For instance, statements related to “brand as a lifestyle beyond fashion products” or “creating deeper customer connections via new touchpoints” were identified as first-order concepts under the broader theme of “Motivations”.

The second phase involved a higher level of abstraction and conceptual consolidation.

The first-order concepts were reexamined and categorized based on conceptual similarities, leading to their organization into “second-order themes”. This step involved a process of recursive abstraction, where granular observations were grouped into more generalized categories that captured underlying patterns. For example, first-order concepts, such as “use of local, organic, or certified ingredients” and “practices like zero-waste or green energy” were consolidated under the second-order themes of “Sustainability ingredients and sourcing” and “Sustainability environmental practices”, respectively. This process was guided by the NVivo Coding Framework developed for this study, which provided a structured yet flexible approach to organizing the qualitative data.

To ensure the trustworthiness and consistency of interpretative decisions, the coding process involved constant comparison and debriefing sessions among the authors. When discrepancies in categorization emerged, they were resolved through collaborative discussion until a consensus was reached on the thematic alignment. This iterative process allowed for a reflexive approach, ensuring that the transition from raw data to higher-level abstraction remained grounded in the informants’ original narratives while minimizing individual bias.

Finally, these second-order themes were further synthesized into “macro-dimensions”, representing the aggregate dimensions of the diversification ecosystem in the luxury brand context.

This consolidation resulted in a structured data representation that aligns with the key dimensions of luxury brand diversification discussed in detail in subsequent sections of this manuscript, particularly reflected in Table 2.

**Table 2:** Data structure.

First-order concepts	Second-order concepts	Aggregate dimensions
Brand as a lifestyle beyond fashion products. Transferring the brand story into culinary and service experiences. Visual and thematic alignment between fashion and restaurant. Use of sound, scent, service and space to create atmosphere. Plans for expansion, scaling or replicability	Lifestyle extension	Motivations
Creating deeper customer connections via new touchpoints. How diners respond to the experience and brand touchpoints. Impact of the restaurant on overall brand perception. Transparency and messaging around sustainability to customers. Training staff on brand values and sustainable practices	Customer engagement	
Exploring new income streams and financial incentives. Differentiating the brand through strategic moves. Use of local, organic, or certified ingredients Practices like zero-waste, LEED certification or green energy. Challenges in aligning fashion logic with food service logic.	Revenue diversification	
Engaging suppliers and partners in sustainability efforts. Collaborating with environmental and social organizations. Incorporating stakeholder feedback into practices. Informing customers about sustainability efforts. Staff training on sustainability.	Stakeholder diversification	Sustainability
Tracking sustainability metrics and progress. Reporting on environmental and social impacts. Assessing the effectiveness of sustainability initiatives.	Measurement and monitoring	
Prioritizing fair trade-certified ingredients. Ensuring ethical labor practices across supply chains. Promoting transparency in sourcing decisions.	Ethical and sustainable sourcing	
Maintaining a uniform visual style between fashion and dining spaces. Using design elements that reflect brand imagery (colors, materials, symbols). Aligning spaces and merchandise to convey a recognizable identity.	Aesthetic consistency	Brand integration
Integrating sounds, scents, lighting and ambiance consistent with the brand. Creating atmospheres that reflect brand values and enhance luxury perception. Transferring brand heritage and story through sensory elements. Unifying multiple touchpoints (visual, digital, culinary) for a cohesive experience.	Multisensory experience	
Telling the brand’s story, values and heritage through menus and touchpoints. Employing strategic storytelling to create emotional connections. Communicating sustainability initiatives and social values as part of the narrative.	Brand narrative	
Resistance within organizational culture. Structural obstacles to change or innovation. Differences in operational logic.	Cultural and structural barriers	Challanges
Limited financial or human resources for sustainability efforts. Challenges in scaling sustainable initiatives. Navigating regulatory constraints. Ensuring compliance with environmental and social standards.	Resource constraints and compliance issues	
Maintaining brand authenticity while innovating. Managing consumer expectations and perceptions. Maintaining authenticity and core values of the brand while embracing new culinary and experiential trends. Aligning the dining experience with the luxury brand’s promise to meet high customer standards.	Balancing brand heritage with innovation	
Strengthening perceptions of a responsible and innovative brand. Enhancing reputation through sustainable practices and experiential offerings. Differentiating from competitors with authentic and valuable propositions.	Brand image	Impacts
Improving satisfaction and loyalty through immersive and coherent experiences. Generating positive feedback and customer engagement across touchpoints. Educating customers on sustainability and social values associated with the brand.	Customer experience	
Differentiating through the association of luxury, sustainability and innovation. Increasing perceived value among customers and stakeholders. Gaining recognition and awards in sustainability and experiential excellence.	Competitive advantage	

Source: Authors’ own elaboration

These macro-dimensions include:

- Strategic motivations for diversification
- Brand integration and experiential cohesion
- Sustainability integration
- Operational challenges in diversification
- Multidimensional impacts of hospitality ventures

After completing first and second-order coding and consolidating aggregate dimensions, we prepared a concise validation package for interviewees, inviting them to verify factual accuracy, interpretive fidelity and the contextualization of exemplar quotations.

To enhance dependability, we conducted an inter‑coder agreement check. Discrepancies were resolved by consensus and the codebook was revised accordingly. We also maintained an auditable trail of coding decisions and memos, and include a reflexivity note to further strengthen transparency.

Specifically, consistent with Gioia qualitative method [80], two researchers independently coded a purposive subset of transcripts and then met for iterative comparison and negotiated‑agreement sessions. Disagreements were examined line‑by‑line, the underlying rationales were recorded in analytic memos and the shared codebook was refined before application to the full corpus. We also held periodic peer‑debriefs to probe rival explanations and to ensure stable use of first‑order concepts, second‑order themes and aggregate dimensions.

The authors’ expertise in the management research domain may orient attention toward strategic and experiential interpretations. To address this issue, we employed reflexive memoing and bracketing throughout all phases of data collection and analysis. A devil’s advocate role was rotated to deliberately surface disconfirming evidence and peer debriefings were conducted, supported by a versioned audit trail documenting coding decisions [81]. A concise reflexivity statement clarified researcher roles, underlying assumptions and methodological safeguards.

## Findings

### Motivations for diversification

A dominant and multidimensional theme that emerged across interviews was the strategic rationale underpinning luxury fashion houses’ diversification into the restaurant and hospitality sector. Rather than viewing the dining venture as a mere commercial expansion, respondents articulated it as a deliberate move to extend brand meaning, strengthen consumer intimacy and express evolving cultural values, particularly in the domains of aesthetics, sustainability and lifestyle consumption. Participants framed the restaurant as a physical and symbolic extension of the brand universe. In this regard, R6 explained:

*We are not just focused on selling clothes anymore. What we want is to create a sense of belonging. In the restaurant, the brand comes through in a different way; it is in the atmosphere, the service and the food.*

This idea of lifestyle extension was echoed by multiple respondents who regarded the hospitality venture as a sensorial translation of the maison’s values and design ethos (R1, R4, R8).

Respondents also emphasized the desire to craft multi-sensory brand experiences that surpass traditional touchpoints like retail stores or runway shows:

*Dining brings together all the senses, what you see, smell, hear, taste and feel. It is a way of telling a story through food* (R5).

Furthermore, as highlighted by R8, the dining experience was perceived not just as atmospheric branding, but as a means to generate affective intimacy

*Luxury clients look for connection. The restaurant gives them a place to pause, take their time and experience the brand in a more personal way.*

While financial performance was acknowledged as an important dimension, some interviewees stressed that return on investment is primarily reputational and emotional. R1 admitted:

*Of course we need to break even, but the real impact is long-term: when a customer brings their family to dine with us every month, they are not just consuming food, they are consuming trust.*

This reframing of financial objectives highlights a more nuanced conceptualization of value creation within the luxury ecosystem.

Another sub-theme under the motivations cluster was strategic differentiation. As the luxury fashion sector becomes increasingly congested with limited editions, collaborations and co-branding initiatives, dining was described as an inimitable frontier, a space less susceptible to replication.

This is pointed out by R6:

*Launching a capsule collection is common but opening a restaurant that truly reflects a century-old brand takes credibility. It shows stability and substance.*

This aligns with the strategic positioning motivation, where brand heritage and authenticity become embodied in curated dining experiences.

Interestingly, for respondents in sustainability roles, diversification into hospitality was seen as a potent instrument to translate the brand’s ESG commitments into practice. Indeed, R3 noted:

*In a flagship store, your footprint is mostly symbolic. But in a restaurant, you touch real systems, food waste, local economies and biodiversity.*

This position was reinforced by R5:

*A restaurant is a living lab for circularity, energy innovation and ethical sourcing.*

Across the sample, a recurring discourse emphasized that luxury dining should not be treated as an opportunistic branding detour, but rather as an architectural component of a long-term cultural strategy. For istance, R1 affimed:

*We never saw it as a trend. For us, it was always about coherence. You enter the restaurant and if we have done it right, you don’t feel like you have stepped away from the brand; you feel like you have gone deeper into it.*

Finally, the motivations for diversification into fine dining among luxury fashion brands reflect a complex interplay of affective, strategic and cultural logics. They represent efforts to deepen consumer engagement through immersive environments, sustain relevance in competitive global markets and materialize intangible brand values, including sustainability, through everyday practices. This convergence of symbolism, experience and strategy positions branded hospitality not as a peripheral experiment, but as a central vector of contemporary luxury branding.

### Sustainability as brand integrity, operational practice and emotional resonance

Another important theme that emerged from the interviews was the importance of sustainability in shaping the identity, operations and experiential design of luxury fashion-branded restaurants. Rather than treating it as an isolated corporate social responsibility obligation, respondents articulated sustainability as an ethical, symbolic and aesthetic imperative, which they saw as an integral part of the maison’s cultural capital and long-term strategic coherence. In this context, sustainability was presented as a lived value touching every aspect of the dining experience, including sourcing, interiors, service rituals and customer perception.

For many participants, sustainability was a means of reinforcing brand authenticity and deepening cultural resonance. As R8 explained:

*We don’t just communicate sustainability; we materialize it. From the reclaimed wood tables to the local wines on the menu, everything must express our values in tangible, visible ways.*

The idea of sustainability as both embedded and performative was echoed by R3, who emphasized the importance of coherence across all brand touchpoints:

*If our garments are made with recycled fabrics and low-impact dyes, but our restaurant serves bottled water from the other side of the planet, we create dissonance. Sustainability must be reflected seamlessly in every aspect of the brand.*

Further, sustainability is conceived as an opportunity for narrative alignment and emotional engagement. For instance, R2 noted:

*Working with local producers is environmentally sound and allows us to tell regional stories that reflect our maison’s roots. When a guest tastes olive oil from the valley where our founder grew up, that’s sustainability as heritage.*

Beyond storytelling, the discussion turned to sustainability as a platform for innovation. R7, described the kitchen as a place for experimentation:

*We treat the restaurant like a test lab. Can we design a tasting menu that is entirely zero-waste? Can we replace imported delicacies with indigenous alternatives without compromising on elegance? These are creative constraints, not limitations.*

However, respondents also acknowledged operational tensions. According to R5:

*There is pressure to be perfect. But in fine dining, expectations are high. Sometimes the most sustainable choice doesn’t meet our aesthetic standards and we have to make compromises.*

Moreover, R1 reinforced this tension between idealism and feasibility:

*We strive for regenerative systems, but some solutions are not yet viable on a large scale. The challenge lies in integrating sustainability without compromising the luxury experience.*

In several cases, sustainability was also presented as a means of achieving sensory and symbolic refinement. For instance, R6 reflected:

*Our guests might not consciously notice every sustainable choice, but they perceive the overall atmosphere: the natural fibres, the absence of waste and the quiet elegance of restraint. This is luxury without excess.*

Interestingly, for some participants, sustainability extended beyond environmental concerns to include a sense of cultural responsibility. As R3 pointed out:

*Younger generations are no longer satisfied to simply consume; they want to feel a sense of purpose and alignment. Our task is to make sustainability aspirational, not didactic. In our context, ethics must be as elegant as the presentation of food.*

Ultimately, sustainability was discussed as a dynamic process of aligning values, systems and aesthetics, rather than a fixed goal. Ensuring that every sensory, material and symbolic element reinforces the brand evolving identity was the purpose behind it. This practice was seen as a way of maintaining brand integrity. As R4 summed up:

*Sustainability is not a merely campaign; it is a way of showing that we care. For the planet, yes, but also for details, community and time. That is what luxury means nowadays.*

Sustainability has emerged as a strategic and sensory lens through which luxury fashion brands interpret and express their values in their restaurants. It functions as a differentiator, a cultural signal and an emotional experience, blending ethics with elegance. In this context, sustainability emerges as a narrative that reinforces the positioning of branded dining as a place where long-term cultural relevance and operational excellence converge.

### Brand integration as narrative consistency and experiential cohesion

A recurring topic that emerged from the interviews was the importance of brand integration, with the restaurant conceived as an authentic continuation of the fashion house’s identity, values and aesthetic principles, rather than functioning in isolation. Respondents consistently describe integration as a deep alignment across sensory, spatial, operational and symbolic dimensions, emphasizing its depth beyond superficial branding. As R6 explained:

*The restaurant reflects the brand. From the way it smells inside to the music at brunch, each detail helps express what the brand stands for.*

R4 also articulated this sense of immersive brand continuity, describing integration as a form of narrative translation.

*What we do is take the codes of the brand. This includes its textures, its colours, its rhythm. We then reinterpret these through food, space and ritual. If we have done it right, guests won’t notice the branding; they will feel the story.*

Moreover, integration involved more than visual or environmental cues, as it demanded alignment in creative philosophy. As R2 and R5 and noted:

*My culinary training was rooted in fine dining, but these days, inspiration comes from unexpected sources, like textiles. When I touch a fabric and it feels raw or grounded, I immediately think of flavors like smoked mushrooms or roasted roots. Cooking becomes a form of storytelling, echoing the aesthetics of the collection through taste.*

*I see my role as curating an experience beyond the plate. Each season, we align our menu with the fashion collection, both visually and conceptually. If the materials speak of warmth or earthiness, our dishes reflect that mood. It is about harmony between cuisine, environment and brand narrative*.

This cross-disciplinary dialogue between fashion and food was pointed out by R4, who emphasized the importance of conceptual coherence:

*The runway and the dining room should feel like chapters of the same novel. Guests should intuitively grasp that they are inside the same imaginative world.*

However, respondents also emphasized the complexity of such integration. As R6, put it:

*You have to respect the brand codes without turning the restaurant into a museum. Integration has to feel natural, not forced. We always ask ourselves: does this choice serve the guest experience or just the brand image?*

Interestingly, some participants viewed brand integration as much a tool for internal alignment as external expression. R3 reflected:

*A culture of consistency is reinforced by the restaurant when it follows the maison’s environmental standards. These standards include reduced energy use, local partnerships and waste tracking. It tells our teams: this is who we are, everywhere.*

Respondents viewed brand integration as a philosophy of coherence. This philosophy requires constant negotiation between the brand’s creative legacy and the operational demands of the hospitality context. As R1 concluded:

*Brand integration is about resonance. Guests should leave feeling not just satisfied, but as if they have entered and briefly lived in the brand’s inner world.*

Brand integration emerges as a strategic imperative that sustains the symbolic integrity of luxury fashion-branded restaurants. It positions the hospitality arm as a meaningful extension of the brand’s identity, where every detail, from the presentation of dishes to the music played, contributes to a coherent, immersive narrative.

### Challenges of translating luxury fashion into hospitality practice

While the concept of luxury fashion-branded restaurants is based on an immersive experience, aesthetic consistency and symbolic depth, respondents often emphasized the complex set of challenges that arise when implementing this concept. These challenges, ranging from organizational misalignment and creative tensions to logistical constraints and sustainability trade-offs, reflect the unique demands of integrating the ethos of high fashion into the realities of the hospitality industry.

A recurring theme across the interviews was the difficulty of merging two distinct industries. As R1 explained:

*Fashion and food operate on different timelines. In fashion, collections change every six months. In hospitality, consistency is key. Finding the right balance between innovation and continuity is an ongoing challenge.*

Similarly, R4 noted the misalignment of creative processes:

*The company wants seasonal concepts that reflect its design language. However, gastronomy has its own logic, involving ingredients, climate and labour. Sometimes the timelines just don’t align.*

Respondents also described cultural and communication differences between the fashion and hospitality teams. R8 claimed:

*There is a learning curve. Our chefs don’t always understand the brand DNA and our fashion designers don’t always grasp operational constraints. We need translators. We need people who can speak both languages.*

One critical challenge was maintaining luxury standards across different regions. As R2 pointed out:

*Our clients expect the same quality regardless of which of our restaurants they visit. However, sourcing local, sustainable ingredients while upholding our aesthetic and culinary standards is incredibly challenging. The key is achieving consistency without losing soul.*

Financial pressures were also mentioned, especially when sustainability was at stake. As R3 reflected:

*Sourcing ethical materials and minimizing waste adds to costs. There is constant pressure to justify these decisions to finance teams who may not fully understand the long-term ESG logic.*

Another recurring point was the complexity of guest expectations. R5 observed:

*Luxury diners are not just there for the food. They want to be surprised, pampered and educated. The illusion is broken if even one detail is off, like the lighting being too cold or the service too slow. Hospitality is brutal in that sense; it goes unnoticed until it fails.*

Despite these obstacles, challenges are considered as necessary growing pains when pioneering a new brand expression format. R1 summarized this sentiment:

*Yes, it is hard. But nobody said that translating a couture philosophy into a living, breathing restaurant would be easy. That is also what makes it meaningful.*

The challenges associated with luxury fashion-branded restaurants highlight the conflict between symbolic ambition and practical execution. From interdisciplinary misalignments to operational complexity and financial scrutiny, the path to brand-aligned hospitality requires ongoing negotiation. However, it is precisely within these frictions that the innovation and uniqueness of this emerging format are forged.

### The multidimensional impacts of branded hospitality initiatives

Interviewees consistently emphasized that the launch and development of luxury, fashion-branded restaurants had an impact that extended far beyond immediate commercial results. The perceived effects were multi-layered, ranging from improved brand storytelling and emotional connection with customers to internal cultural shifts and engagement with external stakeholders. In several cases, participants described these ventures as transformative for both the fashion house and its ecosystem.

From a brand-building perspective, R6 spoke of the powerful amplifying effect of hospitality on brand values:

*The restaurant has become our most emotional brand touchpoint. It is where the abstract becomes real, where guests don’t just see the brand, they taste, hear and feel it.*

This deeper level of engagement was seen as having a long-term impact on our reputation. As R8 noted:

*When guests dine with us and return regularly, it fosters loyalty that retail alone cannot achieve. It is not transactional. It’s about attachment and trust over time.*

From an internal cultural perspective, several respondents described how the hospitality venture created new forms of collaboration and knowledge sharing. In this regard, R3 reflected:

*The restaurant has shifted how we think about sustainability across departments. The fashion department has learned from the food department about sourcing, seasonality and waste, and we are now applying those insights to retail logistics and packaging.*

R1 highlighted the impact on talent development:

*We have attracted chefs who are excited to work in a fashion context and designers who have become fascinated by food. It is cross-pollination. People grow by stepping into unfamiliar territory.*

Although immediate profitability was not always the core focus, some participants reported positive ripple effects on other areas of the business. As R6 pointed out:

*The restaurant is more than just a profit and loss line; it increases footfall to flagship stores, prolongs customer stay and stimulates content creation. It is an ecosystem catalyst.*

The interviews also highlighted institutional and media recognition as part of the impact story. R1 shared:

*When we opened the restaurant, it got more attention than our last two collections. It showed that we are looking at the bigger picture, shaping lifestyles, not only selling clothes.*

Importantly, the symbolic value of the restaurant was emphasized, particularly in terms of positioning the brand as being in tune with culture and forward-thinking. R5 summarized:

*This is about legacy. Fashion is fleeting, but a place where people gather, eat and celebrate leaves a different kind of mark. It becomes part of people’s personal memories.*

The impacts of luxury fashion-branded restaurants were described as relational, reputational and regenerative. They reinforce emotional connections with clients, encourage internal innovation and increase the cultural impact of the brand. Rather than being peripheral, these ventures are seen as dynamic engines of influence within the evolving luxury ecosystem.

## Discussion

The findings of this study reinforce and expand upon the theoretical frameworks on corporate diversification in the luxury fashion sector. Specifically, the strategic diversification of luxury fashion brands into the hospitality and restaurant sector represents a significant evolution in brand architecture.

As claimed by extant literature [1,2,81], diversification operates as a multidimensional strategy enabling long-term growth, brand resilience and stakeholder engagement. Within this perspective, diversification increasingly incorporates CSR as a strategic orientation that connects economic objectives with social legitimacy and responsible value creation for multiple stakeholder groups [23].

In this sense, diversification strategies incorporate CSR principles by using experiential offerings to communicate ethical commitments and socially responsible practices. [82,83].

Actually, the findings of this study reinforce and expand upon these frameworks, showing that the diversification of luxury fashion companies into hospitality, particularly the fine dining sector, is a purposeful initiative aimed at enhancing brand equity through experiential engagement and sustainability signaling.

In line with [31,3], this form of diversification is a deliberate means to embed the brand more deeply within consumers’ lived environments. Unlike conventional extensions, such as cosmetics or eyewear, the integration of hospitality reflects a more profound lifestyle convergence, consistent with research on experiential luxury [58,59].

Indeed, consumers increasingly seek immersive interactions that go beyond symbolic consumption, favoring identity-affirming experiences that offer multisensory depth and cultural relevance. Importantly, these experiences are evaluated in terms of their social and ethical implications, which highlights the importance of CSR in building perceived authenticity and trust [24].

**Fig 1:**
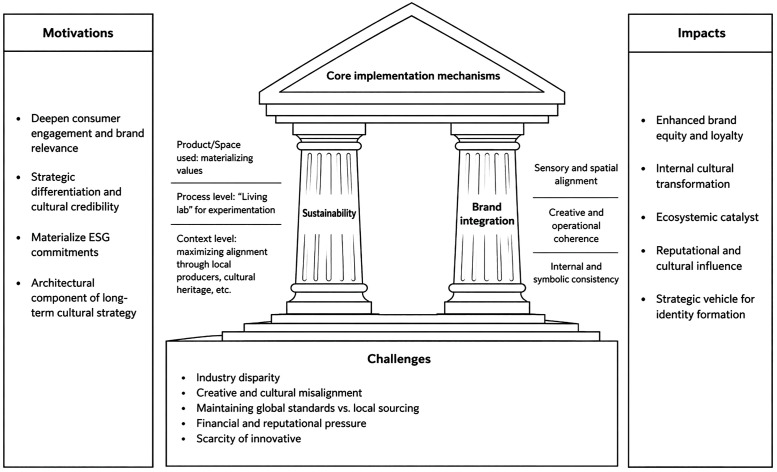
Redifined conceptual framework.

This evolving consumer demand calls for a broader strategic understanding of how luxury fashion brands operationalize diversification into hospitality, particularly through fine dining. Accordingly, Fig 1 offers a conceptual framework of the strategic, operational and outcome-based dimensions underpinning luxury fashion brands’ diversification into sustainable hospitality and restaurant sector. Specifically, the framework outlines a progression from key strategic drivers, such as consumer engagement, cultural differentiation and ESG integration toward two interconnected implementation pillars: sustainability as brand integrity and experiential cohesion through brand integration. These are navigated within a landscape of cross-sectoral challenges, including creative misalignment and operational complexity. The model highlights how, despite these frictions, branded hospitality and restaurant ventures serve as transformative mechanisms that reinforce brand equity, catalyze internal innovation and enhance cultural and reputational capital.

This conceptualization provides a structured lens through which to interpret the five key dimensions that emerged from this study, as outlined in Table 3 and further illustrates how diversification into sustainable hospitality serves not only commercial aims, but also deeper cultural and strategic purposes.

**Table 3:** Key dimensions and practices of diversification ecosystem in the luxury brand.

Dimensions of luxury brand diversification	Strategic objectives and brand value proposition	Key practices and operationalization	Multidimensional impacts and outcomes
**Strategic motivations for diversification**	• A deliberate move to extend brand meaning, strengthen consumer intimacy and express evolving cultural values (aesthetics, sustainability, lifestyle consumption). • Aim to craft multi-sensory brand experiences that surpass traditional touchpoints. • Generate affective intimacy and connection with luxury clients, fostering trust. • Serve as a strategic differentiator, positioning the brand as an inimitable frontier less susceptible to replication, signaling longevity and depth. • Act as a potent instrument to translate the brand’s ESG commitments into practice, functioning as a “living lab” for circularity, energy innovation and ethical sourcing.	• The restaurant functions as a physical and symbolic extension of the brand universe, a sensorial translation of the maison’s values and design ethos. • Dining is leveraged as “edible storytelling” where sight, scent, sound, taste, and texture work together. • Create a “safe space” where consumers can slow down and absorb the brand through rituals. Integrated as an architectural component of a long-term cultural strategy, emphasizing coherence.	• Deepen consumer engagement through immersive environments. • Sustain relevance in competitive global markets. • Materialize intangible brand values, including sustainability, through everyday practices. Fosters long-term loyalty, attachment, and trust, generating reputational and emotional return on investment beyond mere break-even. • Position branded hospitality as a central vector of contemporary luxury branding.
**Brand integration and experiential cohesion**	• Ensure the restaurant is an authentic continuation of the fashion house’s identity, values and aesthetic principles, not functioning in isolation. • Achieve deep alignment across sensory, spatial, operational and symbolic dimensions. • Translate brand codes (textures, colors, rhythm) into food, space and ritual to make guests “feel the story” rather than just noticing branding. • Foster an intuitive understanding that the runway and dining room are “chapters of the same novel”. • Reinforce a culture of consistency internally, following the maison’s environmental standards.	• Every detail, from interior scent to music, food presentation and service rituals, conveys the brand essence. • Culinary interpretations are inspired by fashion elements, e.g., menus designed based on seasonal collections or fabric textures. • Engage in cross-disciplinary dialogue between fashion and food teams. • Constant negotiation to respect brand codes without turning the restaurant into a “museum,” ensuring natural integration that serves guest experience. • Adherence to maison’s environmental standards, such as reduced energy use, local partnerships and waste tracking.	• Creates an immersive brand continuity for consumers. Guests feel they have “gone deeper into” the brand experience. • Prioritizes guest experience and natural integration over forced branding. • Strengthens internal alignment and consistency across departments regarding shared values and standards. • Leads to guests feeling “satisfied” and having “entered and briefly lived in the brand’s inner world”. • Sustains the symbolic integrity of luxury fashion-branded restaurants.
**Sustainability integration**	• Treats sustainability as an ethical, symbolic and aesthetic imperative, integral to the maison’s cultural capital and long-term strategic coherence. • Reinforces brand authenticity and cultural resonance by materializing sustainable values. • Avoids dissonance (e.g., sustainable garments vs. unsustainable restaurant practices). • Creates opportunities for narrative alignment and emotional engagement, linking local sourcing to brand heritage. • Uses the restaurant as a platform for innovation and experimentation in circularity (e.g., zero-waste menus, indigenous alternatives). • Makes sustainability aspirational and an expression of cultural responsibility, where ethics are as elegant as food presentation. • Reflects “care” for the planet, detail, community, and time.	• Sustainability is embedded and performative: evident in reclaimed wood tables, local wines and other tangible elements. • Focus on real systems, such as food waste, local economies and biodiversity. • Work with local producers. • Treat the kitchen as a “test lab” for circularity, experimenting with zero-waste concepts and indigenous alternatives. • Strive for regenerative systems, integrating sustainability without compromising luxury experience, despite aesthetic or viability compromises. • Emphasize “luxury without excess” through natural fibres, absence of waste and quiet elegance.	• Reinforces brand authenticity and deepens cultural resonance. • Enables unique storytelling and connection to regional heritage. • Drives culinary innovation and ethical sourcing practices. • Achieves sensory and symbolic refinement, perceived as “luxury without excess”. Aligns with the preferences of younger, purpose-driven generations. • Strengthens brand integrity and evolving identity. • Functions as a differentiator, cultural signal and emotional experience blending ethics with elegance.
**Operational challenges in translation**	• Acknowledge and navigate the complex demands of integrating high fashion ethos into hospitality realities to ensure effective implementation and avoid missteps. • Recognize the inherent difficulties in merging two distinct industries with different operational logics.	• Managing conflicting operational timelines (fashion’s seasonal changes vs. hospitality’s need for consistency). • Aligning distinct creative processes (fashion design language vs. gastronomy’s ingredient/climate logic). • Overcoming cultural and communication differences between fashion and hospitality teams, requiring “translators” who speak both “languages”. • Sourcing local, sustainable ingredients while maintaining uniform luxury quality standards across diverse global regions. • Justifying higher costs associated with ethical sourcing and waste minimization to finance teams who may prioritize short-term profitability. • Meeting complex and high guest expectations for an immersive, flawless luxury experience beyond just food, where any minor flaw can “break the illusion”.	• Leads to ongoing challenges in balancing innovation and continuity. • Highlights the need for specialized human resources that can bridge industry gaps. • Creates difficulties in achieving global uniformity without compromising local sourcing or authenticity. • Presents financial pressures and internal friction regarding sustainability investments. • Makes hospitality “brutal” in that subtle flaws are noticed and break the luxury perception. • Challenges are reframed as “necessary growing pains” that forge innovation and uniqueness in this emerging format. • Highlights the conflict between symbolic ambition and practical execution.
**Multidimensional impacts of hospitality ventures**	• Achieve impacts extending far beyond immediate commercial results, aiming for transformative effects on the fashion house and its ecosystem, reinforcing emotional connections and cultural influence. • Position the restaurant as a “dynamic engine of influence” within the evolving luxury ecosystem.	• Developing the restaurant as the “most emotional brand touchpoint” where abstract brand values become tangible and multi-sensory (taste, hear, feel). • Fostering long-term customer loyalty and trust that traditional retail alone cannot achieve. • Creating new forms of internal collaboration and knowledge sharing, such as fashion learning from food on sourcing, seasonality and waste and applying these insights to retail. • Attracting and cross-pollinating talent (chefs interested in fashion, designers in food). • Functioning as an “ecosystem catalyst” that increases footfall to flagship stores, prolongs customer stay, and stimulates content creation.	• Has a powerful amplifying effect on brand values and long-term reputation. • Fosters attachment and trust over time. • Shifts internal thinking about sustainability and improves cross-departmental application of insights. • Facilitates talent development and “cross-pollination”. • Generates positive ripple effects on other business areas, contributing to broader business stability and revenue diversification. • Achieves significant institutional and media recognition, positioning the brand as holistic and lifestyle-shaping. • Creates a legacy that becomes part of people’s personal memories, signifying lasting cultural impact. • Described as relational, reputational, and regenerative impacts.

Source: Authors’ own elaboration

Looking at Table 3, with regard to the first dimension, strategic motivations for diversification, the entry of luxury fashion brands into fine dining is driven by a composite of strategic, affective and cultural factors. These ventures aim to consolidate brand presence, reinforce consumer loyalty and differentiate in a saturated luxury market.

While financial performance remains relevant, it is consistently framed as subordinate to more intangible outcomes such as cultural credibility, emotional engagement and the strengthening of long-term consumer relationships. This supports a redefinition of diversification away from purely commercial motivations, aligning instead with the concept of symbolic capital accumulation [5,9].

Furthermore, the hospitality extension functions as a medium for conveying evolving cultural values, such as aesthetics, authenticity and sustainability, which are increasingly central to luxury consumption logics.

In line with the second dimension “branding integration”, ensuring alignment between the brand’s identity and its hospitality expression emerges as a crucial determinant of perceived success. This includes coherence in design language, service rituals and sensorial environments. The goal is to deliver a seamless brand narrative across sectors, preserving the maison’s distinctive codes, while adapting them to the dining context. The concept of “perceived fit” [14,45] is validated in this context, with findings indicating that successful hospitality ventures maintain brand integrity across visual, material and experiential dimensions.

Moreover, the process of integration involves ongoing negotiation between fashion and food professionals to avoid superficial or overly thematic executions, ensuring the restaurant experience functions as a genuine extension of the parent brand.

As regard the third dimension, sustainability in this context is not approached as a reactive compliance mechanism, but rather as an intrinsic brand value with ethical, symbolic and aesthetic implications. Accordingly, these ventures offer opportunities to operationalize ESG commitments in visible, experiential ways, ranging from the use of sustainable materials in interior design to local sourcing and waste reduction in kitchen practices. Such practices reinforce brand authenticity and contribute to the brand’s cultural capital, in line with the findings of [13,15,42]. Additionally, restaurants are used as platforms for sustainable innovation, including experimentation with zero-waste menus, circular food models and regional food narratives, thus transforming abstract commitments into tangible and emotionally resonant practices. These findings echo the argument of [52], who emphasize the role of hospitality as a visible arena for credible sustainability demonstration.

Consistent with the fourth dimension, operational challenges, despite the strategic intent, the integration of luxury fashion into hospitality reveals considerable operational challenges. These include the need to reconcile differing temporalities (e.g., fashion’s seasonality versus hospitality’s continuity), creative methodologies and service expectations.

Knowledge transfer across industries is uneven, and the translation of luxury values into restaurant operations requires significant internal coordination, resource allocation and skill development. Furthermore, maintaining global consistency while adapting to local contexts and sustainability requirements remains a persistent tension, especially when ethical sourcing increases operational complexity. However, these challenges are also perceived as catalysts for innovation, contributing to the uniqueness and authenticity of each venture.

Finally, as for the fifth dimension related to the multidimensional impacts of hospitality venture, restaurants emerge as high-impact touchpoints where brand values are sensorially enacted and emotionally internalized by consumers. This contributes to long-term brand attachment, identity co-creation and relational loyalty, offering dimensions of value that surpass those attainable through traditional retail formats.

Internally, these ventures foster cross-disciplinary learning, facilitate new forms of collaboration and stimulate talent mobility between the fashion and hospitality sectors. They also act as ecosystem catalysts, increasing brand visibility, generating media attention and stimulating footfall to adjacent retail spaces.

Such ventures enhance consumer engagement, institutional and reputational capital, positioning the brand as a holistic lifestyle curator. This broader impact aligns with current academic perspectives on luxury as an evolving cultural system, where meaning is constructed through multi-platform narratives and lived experiences.

Definitively, the diversification of luxury fashion brands into hospitality and restaurant sectors constitutes a transformative strategy that blends brand extension, experiential immersion and sustainability implementation. These ventures offer a compelling case for understanding how symbolic consumption, stakeholder engagement and cultural innovation converge within the contemporary luxury landscape.

The findings contribute substantively to the literature on corporate diversification, experiential marketing and sustainable branding, suggesting that hospitality is not a tangential experiment but a pivotal dimension of twenty-first-century luxury brand strategy.

## Conclusions

This study points out that luxury fashion brands’ diversification into hospitality, specifically into fine dining, operates less as an ancillary profit stream and more as a strategic device for converting brand identity into a lived, multi‑sensory and auditable experience. Drawing on the five aggregate dimensions emerging from the data, namely strategic motivations, brand integration and experiential cohesion, sustainability integration, operational challenges and multidimensional impacts, it highlights that these initiatives consolidate symbolic capital, deepen emotional attachment and enable a tangible ESG commitment. In this sense, restaurants and hotels are high‑impact touchpoints that extend the maison’s cultural project into service environments where aesthetics, authenticity and responsibility must be harmonized in real time.

The main conclusion of the study is that diversification in luxury cannot be reduced to portfolio logic. In the cases analyzed, financial performance is consistently framed as secondary to relational and reputational outcomes, while brand integration emerges as the central condition for success: the closer the translation of the brand’s codes into materials, rituals and narratives, the stronger the perception of coherence and the more durable the consumer–brand bond. At the same time, the cross‑sector move exposes cadence misalignments between fashion’s seasonal creativity and hospitality’s continuous delivery, making knowledge transfer itself a capability to be deliberately designed. Finally, our evidence justifies fine dining as a privileged empirical lens: because it concentrates symbolic density, craft intensity and operational visibility, fine dining makes the mechanisms we theorize (experiential cohesion and the performative nature of sustainability) empirically legible at the point of consumption, thereby allowing rigorous observation of how luxury converts identity into practice.

## Theoretical implications

The study contributes to the literature on management and sustainable luxury by specifying hospitality venues as living laboratories in which sustainability functions simultaneously as brand integrity and emotional resonance. ESG principles are not only communicated but materially enacted through practices, such as local and seasonal sourcing, waste‑aware menu engineering, circular material choices in interiors and staff scripts that embed provenance and responsibility into the guest journey. Such performativity explains why managers prioritize cultural credibility, trust and long‑term loyalty over short‑term margins and why sustainability, when sensorially legible, becomes a distinctive attribute of contemporary luxury rather than a constraint external to the experience.

Moreover, our evidence offers a multifaced view of knowledge‑transfer perspectives in diversification. What transfers most effectively across fashion and hospitality is not first‑order task know‑how but second‑order learning routines (atelier‑style critique, iterative prototyping and boundary‑spanning collaboration) that can be adapted to orchestrate high‑contact service. The documented cadence conflict between seasonal fashion cycles and the continuity imperative of restaurant operations appears as a structural tension that, when actively mediated, produces new capabilities at the intersection of creative direction, operational discipline and sustainability practice. The choice of fine dining accentuates these dynamics: its high controllability and craft intensity make the translation of identity and the ESG effort empirically visible, thereby strengthening the theoretical claim that experiential luxury is best understood where narrative, materiality and performance tightly couple.

## Managerial implications

The results suggest that successful luxury diversification hinges on building formal mechanisms for cross‑sector knowledge transfer, governing sustainable sourcing as a narrative‑risk problem and adopting dual performance logics that align purpose and operations. First, firms should institutionalize boundary‑spanning collaboration between brand and F&B by establishing shared review rituals and joint planning cadences. In practice, translating a seasonal collection into a culinary “capsule” while preserving a stable operational backbone allows the maison to inject creative novelty without compromising service consistency; this approach also reduces the variance in how different locations reinterpret the brand’s codes. By embedding sustainability officers within these routines, knowledge about waste audits, supplier due diligence and material circularity travels more readily from the restaurant to retail logistics and packaging, reinforcing brand integrity across touchpoints. In addition, sustainable sourcing benefits from an approach that distinguishes between iconic anchor ingredients, central to the brand’s heritage narrative and flexible seasonal components that absorb local specificity and climate variability. Such segmentation protects culinary standards and story coherence while improving resilience. Traceability protocols, rigorous supplier onboarding and lot‑level batch tracking equip frontline teams to convert provenance into credible storytelling rather than cosmetic claims. Coupled with menu engineering that elevates preparations with high recovery potential, this approach combines aesthetic expectations with waste reduction in a way that guests can sense without perceiving compromise.

Finally, governance should adopt dual KPIs. Alongside conventional metrics, leadership should monitor experiential cohesion, through mystery‑guest assessments anchored to a concise style and sustainability code and ESG intensity, tracking the share of traceable spend and waste‑per‑cover while linking these to brand outcomes such as flagship footfall, dwell time and user‑generated content sentiment. Regular joint reviews by brand, operations and sustainability teams prevent drift into either surface‑level theming or performative “green” gestures and ensure that the hospitality arm generates equity for the maison by demonstrating responsibility and craft under the most demanding service conditions. Implemented this way, diversification into fine dining improve the brand equity, by consolidating trust, catalyzing internal learning and seeding practices that can be scaled across the broader luxury ecosystem.

## Limits and future research directions

While this study provides valuable and novel insights into the strategic diversification of luxury fashion brands into the hospitality and restaurant sector, it has some limitations. Qualitative interviews were used to gain access to in-depth perspectives from highly experienced professionals, offering privileged insight into strategic and experiential dimensions. However, as with all qualitative research, the findings reflect subjective viewpoints and could be strengthened by broader validation. In this view, future research could adopt mixed or quantitative methods to improve generalizability and evaluate the financial impact of branded hospitality ventures.

Furthermore, cross-cultural or consumer-focused studies could enrich our understanding of how luxury hospitality is perceived and experienced in different markets (e.g., European vs Asian).

Recent evidence from European luxury tourism firms reports a strong and multifaceted commitment to environmental, social and economic sustainability, while complementary results from Asian luxury consumption contexts point to different psychological levers that could translate into distinct expectations in hospitality.

Building on these complementary insights, future research could undertake multigroup survey designs comparing European and Asian guests of branded luxury hotels and restaurants. Such studies should measure beliefs about sustainability (including recycling and circularity), perceptions of certification and standard alignment, perceived price fairness of low carbon or waste reducing options and the perceived fit between sustainability initiatives and brand identity.

Furthermore, experimental work could compare message framings across regions, for example communications emphasizing compliance with recognized sustainability standards versus narratives foregrounding anti-waste benefits, circular menu engineering, or fair pricing of low impact choices and relate these framings to stated willingness to pay, visit intention and perceived brand integrity. Coupling such experiments with qualitative, comparative case studies in different geographical contexts would clarify how brand codes are operationalized in day-to-day ESG practices and which tradeoffs (cost, standardization, narrative coherence) are most salient to managers and guests in each cultural context.

## Supporting information

S1 Appendix Semi-structured interview guide and sources. (DOCX)
