# Supplementary material for: From catwalk to kitchen: A qualitative study of luxury fashion companies’ diversification into hospitality and restaurant sector
Source: PLoS One. 2026 Jul 1;21(7):e0350846. doi: 10.1371/journal.pone.0350846 (PMC13322538; doi:10.1371/journal.pone.0350846)
Supplement: S1 Appendix — (DOCX) [file pone.0350846.s001.docx]

**S1 Appendix. Semi-Structured interview guide and sources**

This semi-structured interview protocol reproduces the guide used to collect data for the study. It is organized by sections that map to the aggregate dimensions in the Gioia data structure (strategic motivations; brand integration and experiential cohesion; sustainability integration; operational challenges; multidimensional impacts). Items are tagged as follows: [L] = derived from literature, [A] = developed by authors for this study, [E] = emergent probe used inductively. The complete question list is provided to enhance transparency and assessability.

**Legend**

[L] Literature-derived [A] Author-developed [E] Emergent probe

**Minimal data capture sheet**

- Interview ID; Date; Mode (video/phone); Duration (minutes)
- Informant Role and function (Top management / F&B / R&D / Sustainability / Marketing)
- Years in role; Geography/Market scope
- Venue type (restaurant/café/hotel); City
- Consent recorded (Y/N)

**Intro, consent and background**

1. Confirm informed consent for recording and anonymization (verbal informal consent)

2. Please describe your current role, unit, tenure and scope.

3. Briefly outline the brand’s hospitality venture(s): format, launch year, key partners (e.g., chef).

**Section 1. Strategic motivations for diversification**

1.1 What initial strategic motivations led your company to enter hospitality/fine dining? [L]

1.2 Beyond financial outcomes, what brand-level objectives (e.g., cultural positioning, emotional intimacy, stakeholder engagement) were prioritized? [L]

1.3 How important was perceived fit between the parent brand and the hospitality venture in gaining internal/external buy-in? [L]

1.4 Which alternative routes (e.g., collaborations, pop-ups) were considered and why was a permanent hospitality format chosen? [A]

Probes (examples): “What would have happened if you had not launched the restaurant?” [E]

**Section 2. Brand integration & experiential coherence**

2.1 How do brand codes (materials, colors, narratives) translate into spatial design, menu, service rituals, and music? Please give concrete instances. [L]

2.2 How do you ensure ongoing coherence between fashion collections and the hospitality experience (e.g., seasonal storytelling)? [A]

2.3 Describe a moment when brand integration felt “forced” and how you corrected it. [A]

2.4 To what extent do parent brand equity and extension fit guide decisions (naming, chef partnerships, pricing)? [L]

Probe (example): “Walk me through the last menu change—who approved what, when and why?” [E]

**Section 3. Sustainability (ESG) integration**

3.1 Which ESG principles were embedded by design (e.g., sourcing, zero-waste, energy, water, labor, community)? [L]

3.2 How do you balance aesthetics and luxury cues with sustainability requirements (e.g., look/feel of recycled materials)? [L]

3.3 How is sustainability communicated to guests (whisper vs. shout)? What have you learned about skepticism/credibility? [L]

3.4 Give an example of using the restaurant as a “living lab” (circular pilots, local substitutions, zero-waste menu). [A]

Probe (example): “If the CFO cut the ESG budget by 30%, what would you protect first, and why?” [E]

**Section 4. Operational challenges & knowledge transfer**

4.1 Where did you experience the biggest frictions between fashion and hospitality logics (timelines, seasonality, training, suppliers)? [L]

4.2 Which capabilities from fashion transferred well, and which did not (where did you have to build new know-how)? [L]

4.3 How do you pursue global consistency while adapting to local sourcing/ESG constraints? [A]

Probe (example): “Tell me about the last time a sustainability goal clashed with guest expectations—what trade-off did you make?” [E]

**Section 5. Outcomes & impact**

5.1 What brand outcomes do you observe (loyalty, attachment, word-of-mouth, flagship spillovers, media)? [L]

5.2 What internal effects have emerged (cross-functional learning, talent attraction, sustainability spillovers to retail/packaging)? [A]

5.3 Looking ahead, what would you do differently in the next hospitality venture? [A]

Probe (example): “Describe a guest moment that convinced you the venture is (or is not) delivering the intended brand experience.” [E]

**Section 6. Closing**

6.1 Is there anything we did not ask that is crucial to understand luxury hospitality by fashion brands? [A]

6.2 Whom else should we interview (snowball)? [A]

**S1 Table.** Mapping to aggregate dimensions

| **Aggregate dimension** | **Section(s) of guide** | **Items** |
| --- | --- | --- |
| Strategic motivations for diversification | Section 1 | 1.1–1.4 |
| Brand integration & experiential cohesion | Section 2 | 2.1–2.4 |
| Sustainability integration | Section 3 | 3.1–3.4 |
| Operational challenges in diversification | Section 4 | 4.1–4.3 |
| Multidimensional impacts of hospitality ventures | Section 5 | 5.1–5.3 |

Source: Authors’ own elaboration

**Short consent script**

“Thank you for your time. With your permission, we will audio-record this interview to ensure accuracy. Your participation is voluntary; you may skip any question or stop at any time. Your responses will be anonymized; we will not attribute quotes to you or your company without explicit permission. Do we have your consent to proceed and record?”

**References**

Athwal N, Wells VK, Carrigan M, Henninger CE. Sustainable luxury marketing: a synthesis and research agenda. Int J Manag Rev. 2019;21(4):405-26.

Chen C, Kim EL, Schuckert M. Haute couture-to-table? A study of luxury fashion-brand restaurants/cafés. J Glob Scholars Mark Sci. 2023;33(2):312-25

Gioia, D. A., Corley, K. G., & Hamilton, A. L. (2013). Seeking Qualitative Rigor in Inductive Research: Notes on the Gioia Methodology. Org Res Meth, 16(1), 15–31.

Hafner C. Diversification in family firms: a systematic review of product and international diversification strategies. Rev Manag Sci. 2021;15(3):529-72.

Kallio, H., Pietilä, A.-M., Johnson, M., & Kangasniemi, M. (2016). Systematic methodological review: Developing a framework for a qualitative semi-structured interview guide. J. Adv Nurs, 72(12), 2954–2965.

Peng, C., Bijmolt, T. H. A., Völckner, F., & Zhao, H. (2023). A Meta-Analysis of Brand Extension Success: The Effects of Parent Brand Equity and Extension Fit. J Mark, 87(6), 906–927.

Sepe F, Muto V, Prisco A, Tani M. Fashion and sustainability: Evidence from the consumption of second‐hand clothes. Corp Soc Responsib Environ Manag. 2025;32(1):947-62.

Shahid S, Paul J, Gilal FG, Ansari S. The role of sensory marketing and brand experience in building emotional attachment and brand loyalty in luxury retail stores. Psychol Mark. 2022;39(7):1398-412.
